# One-pot synthesis of VO _x_ /Al _2_ O _3_ as efficient catalysts for propane dehydrogenation

**DOI:** 10.3906/kim-1907-53

**Published:** 2020-02-11

**Authors:** Guangjian WANG, Hao XU, Kai LU, Zhihao DING, Liancheng BING

**Affiliations:** 1 School of Chemical Engineering, Qingdao University of Science and Technology, Qingdao P.R. China

**Keywords:** One-pot synthesis, propane dehydrogenation, VO _x_ /Al _2_ O _3_ catalysts

## Abstract

Vanadium oxides, as highly efficiently catalysts, are widely applied in various catalytic reactions, such as the dehydrogenation of light alkanes and epoxidation of alkenes. In this paper, a series of VO _x_ /Al _2_ O _3_ catalysts were fabricated by the 1-pot method for catalytic propane dehydrogenation. The results indicated that the VO _x_ /Al _2_ O _3_ catalysts with loading of 10 wt.% vanadium exhibited optimized catalytic performance. The as-prepared catalysts were characterized by N _2_ adsorption-desorption, XRD, TEM, H _2_ -TPR, and XPS to explore the texture properties, morphology, and electronic environment of vanadium. In addition, several vanadium catalysts were also prepared by the incipient wetness impregnation (IWI) method to compare their catalytic performance with the 1-pot synthesized catalysts. The catalysts synthesized by the 1-pot method exhibited higher selectivity of propylene and longer catalyst lifetime at high propane conversion when compared to the counterpart synthesized by the IWI method.

## 1. Introduction

Propylene is a fundamental and key feedstock for the production of important chemical intermediates such as polypropylene, acrylonitrile, and 2-propanol [1]. The production of propylene from catalytic propane dehydrogenation (PDH) using platinum (Pt)- or chromium oxide (CrOx) -based catalysts is practiced commercially, but the restructuring of Pt and CrO _x_ species is also a great challenge for the stability of catalysts after regeneration [2–4]. Moreover, Pt- or CrO _x_ -based catalysts are limited because Pt-based catalysts employ noble metal, while CrO _x_ -based catalysts create pollution of the environment [5,6]. Vanadium, as a cost-effective and environmentally friendly metal, is an attractive alternative catalyst for PDH [7]. Although vanadium catalysts are mainly applied in the oxidative dehydrogenation of propane, researchers have found that they also displayed excellent catalytic performance and high stability for PDH [8,9]. For instance, Gong et al. found that VO _x_ /Al _2_ O _3_ catalysts prepared by the incipient wetness impregnation (IWI) method exhibited extremely high selectivity for propylene (80%) with 35% conversion of propane [5]. The surface-bound hydroxyl groups on VO _x_ /Al _2_ O _3_ played an important role in catalytic performance for PDH. Ovsitser et al. reported the synthesis of VO _x_ /Al _2_ O _3_ by the IWI method for PDH with 80% propylene selectivity and 45% propane conversion [10]. Sokolov et al. found that VO _x_ -supported MCM-41 exhibited high stability for PDH, being more stable than Pt/MCM-41 or CrO _x_ /MCM-41 catalysts [11]. Yan et al. found that the crystalline temperature of aluminum had great influence on the catalytic performance of VO _x_ /Al _2_ O _3_ catalysts, and catalysts with loading of 10 wt.% vanadium displayed high propane conversion and propylene selectivity [12]. Despite the enhancement of catalytic performance, these catalysts, prepared by the multistep synthetic method, normally result in the poor dispersion of active metal [13].

It is worth noting that the superior catalytic performances of 1-pot synthesized metal catalysts were proved via numerous related studies due to the good dispersion and sintering resistance of active metals [14,15]. The dispersion of active metal and strong metal-support interaction dramatically enhanced the catalytic efficiency of catalysts [16]. Significant progress has been made in metal or metal oxide catalysts synthesized by the 1-pot method with the incorporation of various metals, including Pt, Pb, Ni, and Mo [17–20]. For instance, Guo et al. used VO _x_ /SiO _2_ catalysts synthesized by the 1-pot method to obtain a high propane conversion of 45% [21]. Chu and Luo reported PtSn/Mg-SBA-15 catalysts that achieved high catalytic performance for PDH [22]. Filez et al. synthesized Pt-based catalysts by the 1-pot method that achieved superior catalytic performance for PDH [23]. To date, however, there have been no reports on the synthesis of VO _x_ /Al _2_ O _3_ catalysts via the 1-pot method.

In this paper, we present a facile 1-pot method to synthesize VO _x_ /Al _2_ O _3_ catalysts, which are highly efficient, cost-effective, and environmentally friendly. Unlike other VO _x_ /Al _2_ O _3_ catalysts prepared by the multistep synthetic method, good dispersion of the active metal (VO _x_ ) can be obtained during the formation of catalysts. The VO _x_ /Al _2_ O _3_ catalysts were synthesized through the 1-pot method combined with evaporationinduced self-assembly (EISA) using sodium carboxymethyl cellulose (CMC) as an anionic surfactant [24–26]. As can be seen in the Scheme, the introduction of vanadium species was achieved by adding the solution of aluminum nitrate and CMC into the solution of the vanadium precursor. Next, aqueous ammonia was added to enhance the assembly by promoting the interaction between the vanadium precursor and CMC. Moreover, aluminum oligomer, derived from aluminum nitrate during hydrolysis, can interact with the CMC by hydrogen bonding, ensuring assembly with the vanadium precursor. In short, the alumina and vanadium oxide precursors were mixed in solution state and CMC was used as an anionic surfactant. The vanadium precursor dispersed uniformly in the framework of alumina gel during the evaporation self-assembly process and eventually formed a vanadium-doping disordered Al _2_ O _3_ catalyst. Meanwhile, a series of VO _x_ /Al _2_ O _3_ catalysts were also synthesized by the IWI method to compare the catalytic performance with that of the VO _x_ /Al _2_ O _3_ catalysts synthesized by the 1-pot method. It was found that the catalysts prepared by the 1-pot method exhibited higher catalytic performance and stability than those prepared by the IWI method, and the O-10-VO _x_ /Al _2_ O _3_ catalysts displayed optimized catalytic performance. In addition, properties of the catalysts were investigated by N _2_ adsorption-desorption, XRD, TEM, H _2_ -TPR, and XPS for a better understanding of catalytic behavior.

**Scheme:**
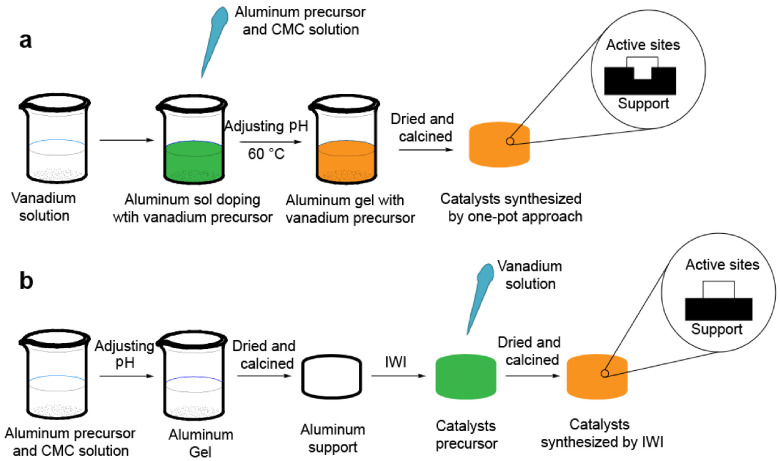
One-pot (a) and IWI (b) methods for preparing the VO _x_ /Al _2_ O _3_ catalysts.

## 2. Experimental

### 2.1. Catalyst synthesis

#### 2.1.1. One-pot method

One-pot synthesized VO _x_ /Al _2_ O _3_ catalysts, with loading of 10 wt.% vanadium, were prepared via the 1-pot method combined with EISA. A specific amount of ammonium metavanadate and oxalic acid was dissolved at 60 ◦ C using a beaker in 160 mL of deionized water to form a clear solution. The molar ratio of ammonium metavanadate and oxalic acid was 1:1. The solutions of aluminum nitrate and CMC were subsequently added to the solution of ammonium metavanadate under vigorous stirring. Aqueous ammonia was then dropped into the mixture until the pH value was modified to 8, followed by stirring at 60 ◦ C for 8 h. At the end of stirring, the green precipitations were dried in an oven at 100 ◦ C for 12 h. Next, the precursors were calcined at 550 ◦ C for 10 h to obtain the O-10-VO _x_ /Al _2_ O _3_ catalysts, where O represents the 1-pot synthesis. Other catalysts synthesized by the 1-pot method were prepared with different vanadium contents.

#### 2.1.2. Incipient wetness impregnation method

In a typical process, a specific amount of ammonium metavanadate and oxalic acid was dissolved at 60 ◦ C using a beaker in 60 mL of deionized water to form a clear solution. The molar ratio of ammonium metavanadate and oxalic acid was 1:1.

To synthesize mesoporous aluminum, CMC was subsequently added to the solution of aluminum nitrate under vigorous stirring. The pH of the solution was then modified from 2 to 8 at 60 ◦ C under stirring to form uniform precipitates. The uniform precipitates were dried in an oven at 100 ◦ C for 12 h and then calcined in a muffle furnace at 550 ◦ C for 10 h, and the obtained mesoporous aluminum was used as supports.

The catalysts, with loading of 10 wt.% vanadium, were prepared by the IWI method. The mesoporous aluminum was impregnated with metavanadate solution and then dried at 100 ◦ C for 12 h. The catalysts were subsequently calcined at 550 ◦ C for 10 h. The resulting catalysts were denoted as I-10-VO _x_ /Al _2_ O _3_ , where I represents the incipient wetness impregnation method. Other impregnation catalysts were prepared with different vanadium contents.

### 2.2. Characterizations

N _2_ adsorption-desorption isotherms of all of the samples were obtained with an Autosorb-1 (Quantachrome Instruments Inc., Boynton Beach, FL, USA). The surface areas and total pore volumes of the catalysts were obtained by the Brunauer–Emmett–Teller (BET) method and the N _2_ adsorption amount was at a relative pressure of P/P0 = 0.99. The size distribution curves of the mesopores and micropores were determined from the adsorption-desorption isotherms using the Barrett–Joyner–Halenda and Horvath–Kawazoe methods, respectively. X-ray powder diffraction (XRD) spectra were obtained with an X’Pert PRO MPD diffractometer (Malvern Panalytical Ltd., Malvern, UK) with Cu Kα radiation (λ = 0.1542 nm). H _2_ -TPR curves of the samples were collected on an Autosorb-iQ (Quantachrome Instruments) by a thermal conduction detector. Transmission electron microscopic (TEM) images were obtained using a JEM-2010 microscope (JEOL Ltd., Akishima, Tokyo, Japan) at an accelerating voltage of 200 kV. X-ray photoelectron spectroscopy (XPS) measurements were carried out by a Thermo Fisher ESCALAB 250 X-ray photoelectron spectrometer (Waltham, MA, USA) equipped with an A1 Kα X-ray source.

### 2.3. Catalytic performance

Catalytic reactions were performed in a continuous flow evaluation device, which included a mass flow controller, stainless steel reactor (10 nm), exhaust gas treatment unit, and analysis system. Under a N _2_ flow of 30 mL/min, the stainless steel reactor loading samples of 3 g was heated from 20 ◦ C to 550 ◦ C within 3 h. Next, the N _2_ flow was replaced by a H2 flow of 5 mL/min to reduce the catalysts for 8 h at 550 ◦ C. After that, the temperature was increased to 610 ◦ C and the H2 flow was closed. Next, a propane flow of 60 mL/min was introduced into the reactor, and the products were analyzed online by an Agilent 7820A GC (Santa Clara, CA, USA).

X (%) = ([N _C3H8_ ] _in_ -[N _C3H8_ ] _out_ ) / [N _C3H8_ ] _in_

Y (%) = ([N _C3H6_ ] _out_ -[N _C3H6_ ] _in_ ) / [N _C3H8_ ] _out_

S (%) = ([N _C3H6_ ] _out_ -[N _C3H6_ ] _in_ ) / ([N _C3H8_ ] _in_ -[N _C3H8_ ] _out_ )

The above formulas were used to analyze the results of the experiment, where the conversion of propane and the yield and selectivity of propylene are represented by X, Y, and S, respectively. N _C3H8_ and N _C3H6_ are the number of carbon atoms, where C _3_ H _8_ and C _3_ H _6_ represent propane and propylene, respectively.

## 3. Results and discussion

### 3.1. Catalytic performance

**Figure 1:**
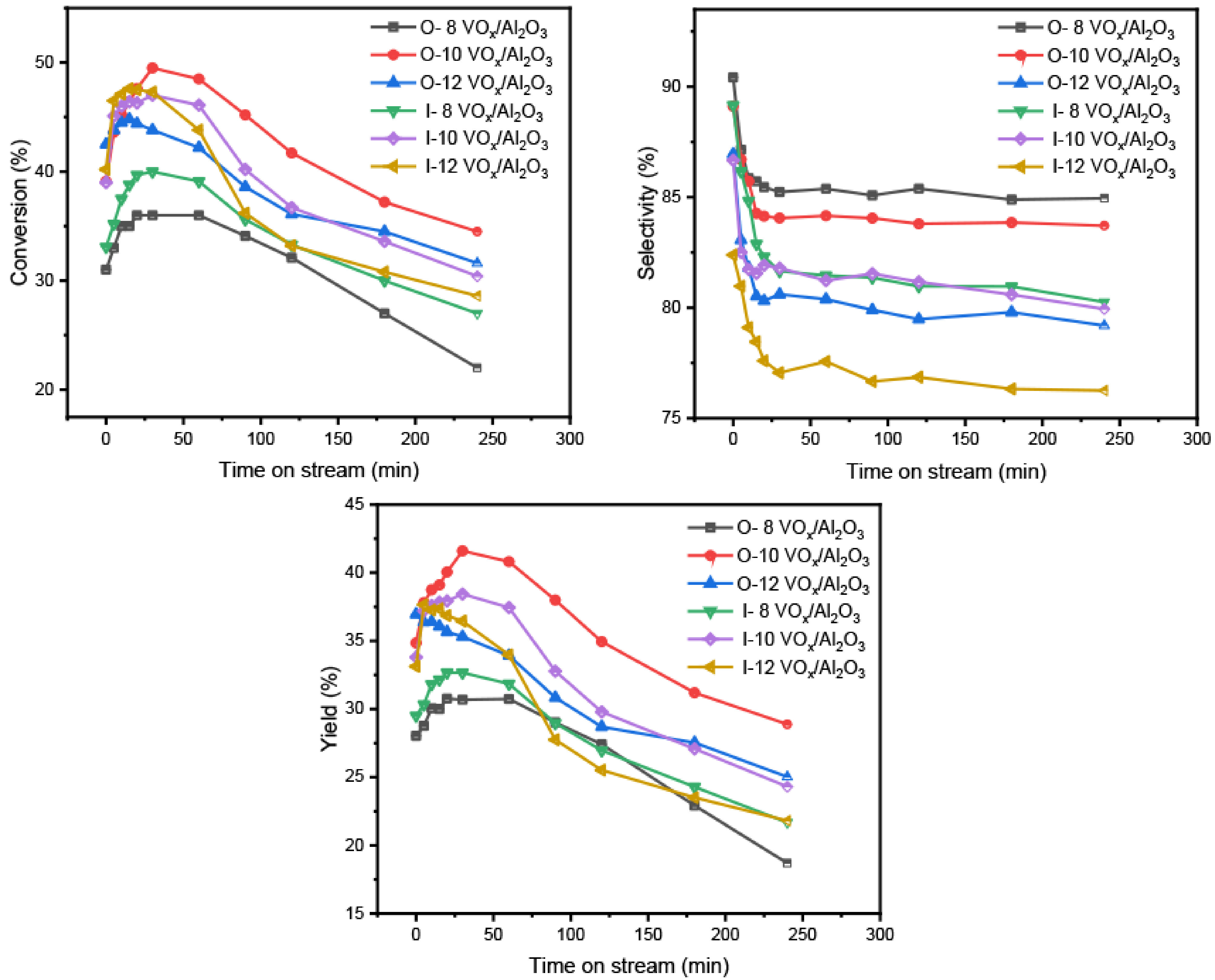
Catalytic performance of the vanadium catalysts prepared by one-pot method and IWI method.

Catalysts prepared by different methods were investigated to obtain the catalytic performance for PDH and the results are shown in Figure 1. It can be seen that the conversion of propane and yield of propylene increased rapidly during the initial 30 min for all of the catalysts and then decreased slowly after reaching the peak, which may have been due to the formation of carbon coating on the active sites [12]. At the same time, the selectivity of propylene was stable after a sharp drop. Among the 3 catalysts synthesized by the 1-pot method, the O- 10-VO _x_ /Al _2_ O _3_ catalysts showed the highest catalytic performance with an optimized propane conversion of 50% and propylene selectivity of 85% at 1 h of PDH reaction, while the O-8-VO _x_ /Al _2_ O _3_ catalysts exhibited the poorest catalytic activity. For the O-10-VO _x_ /Al _2_ O _3_ catalysts, the propane conversion and propylene selectivity fluctuated between 35% and 50% and between 84% and 89%, respectively. This may have been due to the deactivation of active sites caused by carbon deposition.

When compared with the VO _x_ /Al _2_ O _3_ catalysts synthesized by the IWI method, the inactivation rate of those synthesized by the 1-pot method was slower, indicating that the catalysts synthesized by the 1-pot method had a longer catalytic lifetime than those synthesized by the IWI method [27]. In addition, after 4 h of reaction, the O-10-VO _x_ /Al _2_ O _3_ catalysts still displayed the best catalytic activity, while the I-10-VO _x_ /Al _2_ O _3_ catalysts had poor catalytic activity. This further demonstrated that the catalysts prepared by the 1-pot method had higher catalytic performance for PDH. Moreover, the turnover frequency (TOF) values of the as-prepared catalysts in PDH were calculated by TOFi = MVXin0 i mcatcV to investigate the catalytic activity of the catalysts, where MV , Xi , n0i, mcat , and cV are the atomic mass of vanadium (50.94 g/mol), conversion of component i, molar flow rate of component i (mol/s), catalyst mass (g), and mass fraction of vanadium doping in the catalysts, respectively [2]. The results are shown in Table S1 and the O-10-VO _x_ /Al _2_ O _3_ catalysts still exhibited the highest catalytic activity. A summary of the catalytic data of the PDH catalysts is summarized in Table 1. When compared with the vanadium-based catalysts available in the recent literature, the propane conversion of the O-10-VO _x_ /Al _2_ O _3_ catalysts synthesized herein was higher than that of the VO _x_ /Al _2_ O _3_ catalysts prepared by the IWI method under similar reaction conditions, which was related to the high dispersion of vanadium and stable active sites. Although the propane conversion and propylene selectivity were similar to those of the VO _x_ /SiO _2_ catalysts, the space velocity of the O-10-VO _x_ /Al _2_ O _3_ catalysts was higher. When compared with Pt and CrO _x_ catalysts, the catalytic activity of the O-10-VO _x_ /Al _2_ O _3_ catalysts for PDH was higher than that of the CrO _x_ Na/Al _2_ O _3_ catalysts, which was similar to that of the Pt catalyst [28,29]. Moreover, the catalysts prepared herein were efficient, cost-effective, and environmentally friendly.

**Table 1:** Summary of the catalytic data of the dehydrogenation catalysts.

Catalysts	Active metal	Temperature	Propane conversion (2 h)	Propylene selectivity (2 h)	WHSV (h ^-1^ )	Refs.
^a^ VOx/Al _2_ O _3_	VO _x_	610 ◦C	42%	85%	1.2	This work
VO _x_ /SiO _2_	VO _x_	580 ◦C	45%	90%	0.3	2
VO _x_ /Al _2_ O _3_	VO _x_	600 ◦C	25%	79%	8	5
VO _x_ /MCM-41	VO _x_	600 ◦C	45%	80%	0.48	11
CrO _x_ Na/Al _2_ O _3_	CrOx	550 ◦C	42%	80%	0.12	29
Pt-Sn/Al _2_ O _3_	Pt	590 ◦C	45%	96%	5.2	28

### 3.2. BET

**Figure 2:**
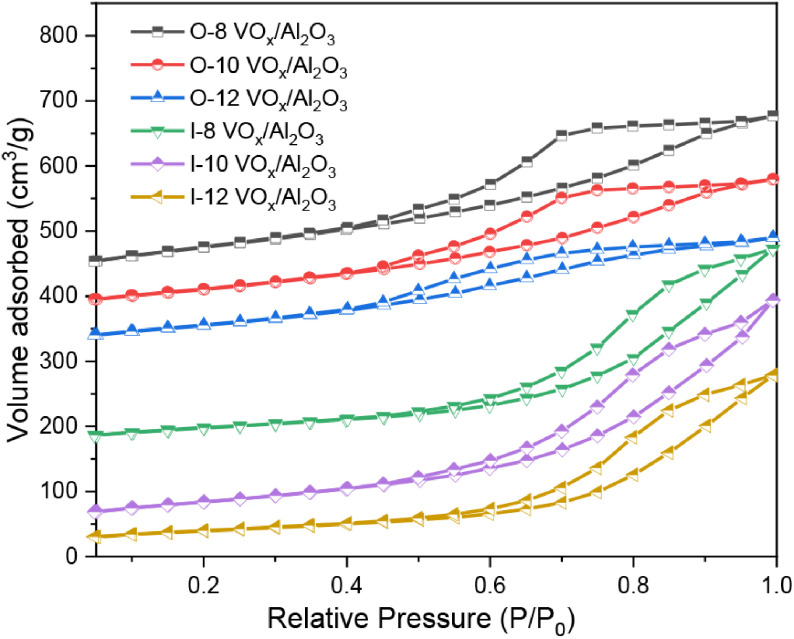
N _2_ adsorption-desorption isotherms of the catalysts.

N _2_ adsorption-desorption was performed to analyze the detailed textural properties of the as-prepared catalysts. For the catalysts synthesized by the 1-pot method, catalysts with different vanadium doping presented similar N _2_ adsorption-desorption isotherms (Figure 2), which were type-IV isotherms with H-2 hysteresis loops according to the IUPAC classification, implying that they had an ink-bottle mesoporous structure. Meanwhile, the catalysts synthesized by the IWI method exhibited a type-IV isotherm with H-1 hysteresis loops, suggesting that the synthetic method could influence the mesoporous structure of γ -Al _2_ O _3_ . As can be seen in Table 2, the catalysts synthesized by the 1-pot method had a higher specific surface area and pore volume, which may have been due to the interaction between the vanadium precursor as well as the change of the weak interaction between the alumina precursor and CMC [30]. Moreover, the larger specific surface area increased the access between propane and the active sites, leading to higher catalytic performance of the catalyst for PDH. In addition, increased vanadium doping led to a decrease in the specific surface area and pore size, indicating that the VO _x_ species were located in the channel of γ -Al _2_ O _3_ [2]. Moreover, a decrease in the hysteresis loop length of the samples was observed with increasing vanadium incorporation, simultaneously accompanied by decreases in the pore volume, which indicated that the amount of vanadium doping could remarkably affect the texture properties of catalysts and correspondingly affect the catalytic performance of the catalyst. The O-10-VO _x_ /Al _2_ O _3_ catalysts had a proper pore structure, specific surface area, and vanadium content, which resulted in optimized catalytic performance.

**Table 2:** Textural properties of the as-prepared catalysts.

Catalysts	SBET (m2/g)	DBJH (nm)	Vt (cm3/g)
O-8-VO _x_ /Al _2_ O _3_	276	17.1	0.36
O-10-VO _x_ /Al _2_ O _3_	224	18.1	0.31
O-12-VO _x_ /Al _2_ O _3_	209	19.2	0.26
I-8-VO _x_ /Al _2_ O _3_	198	18.1	0.30
I-10-VO _x_ /Al _2_ O _3_	175	19.2	0.28
I-12-VO _x_ /Al _2_ O _3_	162	20.1	0.23

### 3.3. XRD

**Figure 3:**
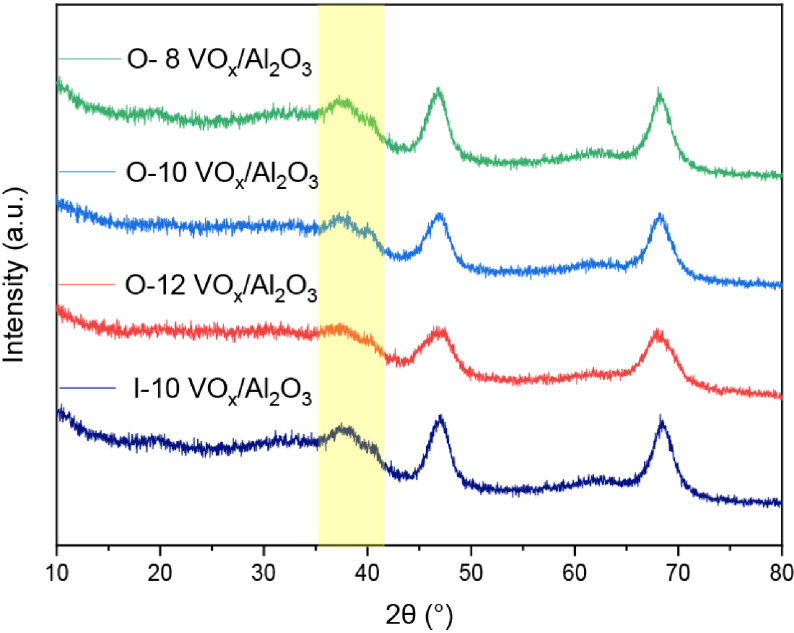
XRD patterns of the catalysts synthesized using the 1-pot method and I-10-VO _x_ /Al _2_ O _3_ catalysts.

The powder XRD patterns of the catalysts loaded with different vanadium contents are shown in Figure 3. For the catalysts synthesized by the 1-pot method, the broad peaks at 45.8◦ and 66.8◦ can be assigned to the structure of γ -Al _2_ O _3_ . There was no observable peak attributable to crystalline V _2_ O_5_ for any of the catalysts with vanadium loading below 12 wt.%, which can be ascribed to high dispersion, and the size of the vanadium species was less than 2 nm. For the I-10-VO _x_ /Al _2_ O _3_ , no observable peak of VO _x_ was detected, indicating that there was no vanadium oxide on the catalysts prepared by the IWI method. Interestingly, the characteristic peak at 37.5◦ assigned to Al _2_ O _3_ gradually decreased with increased vanadium incorporation. In contrast, the peak was significantly clearer in I-10-VO _x_ /Al _2_ O _3_ , despite having the same vanadium content as the O-10-VO _x_ /Al _2_ O _3_ catalysts, which may have been due to the stronger interaction between the VO _x_ and supports.

### 3.4. TPR

**Figure 4:**
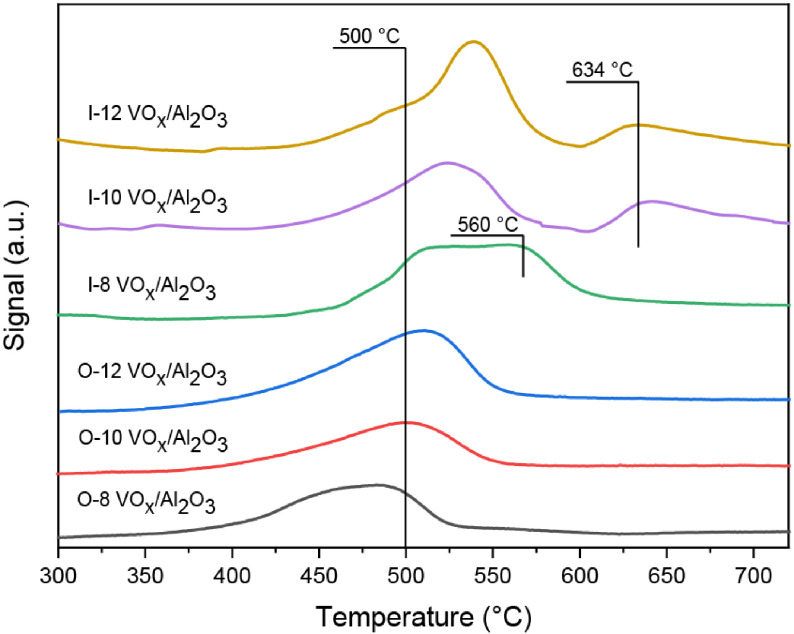
H _2_ -TPR profile of the catalysts.

To determine the reducibility and stability of the as-prepared catalysts, H _2_ -TPR measurements were performed. The TPR patterns of all of the catalysts between 300 and 720 ◦ C are shown in Figure 4. The reduction peaks in the range of 490–510 ◦ C of the catalysts prepared by the 1-pot method can be assigned to uniformly monomeric and/or low polymeric vanadium species, indicating that the active sites were anchored on the support stably [31–33]. When compared with the catalysts synthesized by the 1-pot method, the reduction peak of the catalysts synthesized by the IWI method slightly shifted to a high temperature, suggesting that the dispersion of vanadium on the catalyst prepared by the IWI method was worse. A shoulder peak at 560 ◦ C was observed in the I- 8-VO _x_ /Al _2_ O _3_ catalysts, which may be attributed to the aggregation of vanadium species [2]. Meanwhile, additional reduction peaks at 634 ◦ C in the I-10-VO _x_ /Al _2_ O _3_ and I-12-VO _x_ /Al _2_ O _3_ catalysts were observed, which may have been due to the presence of V _2_ O_5_ crystallites [21]. By combining the BET and XRD analyses, it can be concluded that the catalysts prepared by the 1-pot method presented higher dispersion of vanadium and more stable active sites. Previous literature has shown that monomeric and/or low polymeric vanadium species are the main active species for PDH reaction [31]. Hence, the catalysts prepared by the 1-pot method exhibited higher catalytic performance and stability than those prepared by the IWI method for PDH.

### 3.5. TEM and XPS

**Figure 5:**
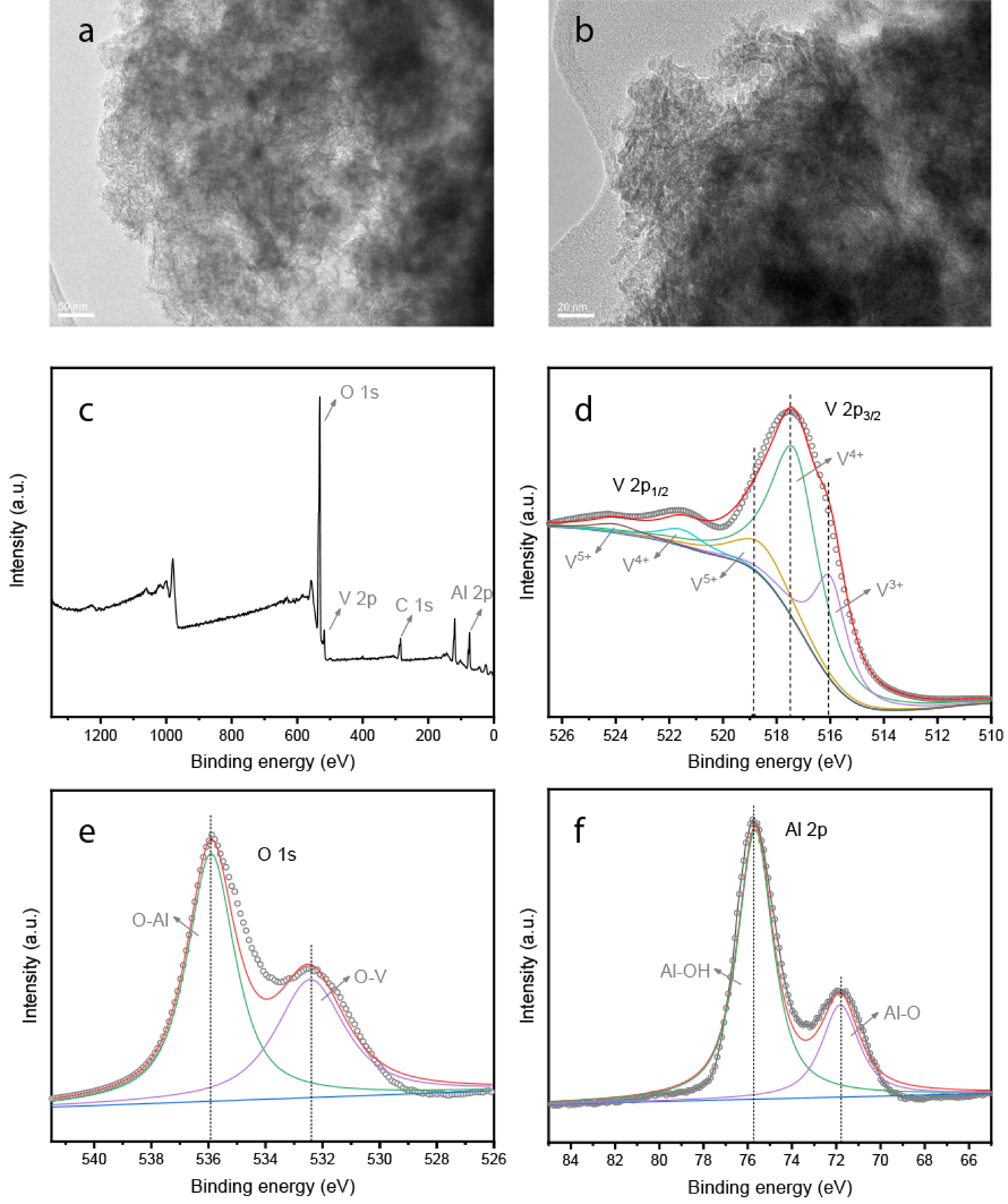
TEM images and XPS spectra of the as-prepared catalysts (a), TEM image of O-10-VO _x_ /Al _2_ O _3_ (b), XPS spectra of O-10-VO _x_ /Al _2_ O _3_ (c), V 2p (d), O 1s (e), and Al 2p core-level spectra (f).

To investigate the morphology of the catalysts and dispersion of the vanadium species, TEM characterization was performed for the O-10-VO _x_ /Al _2_ O _3_ catalysts. Figures 5a and 5b display the TEM images of the O-10-VO _x_ /Al _2_ O _3_ catalysts. The catalysts exhibited a disordered worm-like channel, in which the fine stripes represented the channel network and the dark areas represented the alumina matrix. Figure S1 shows the HRTEM image of the catalyst. No V _2_ O_5_ crystallites were found, indicating that the vanadium species were mainly present as oligomers on the catalyst, which was consistent with the results of the XRD and H _2_ -TPR.

The element bonding configuration of the O-10-VO _x_ /Al _2_ O _3_ catalysts was investigated by XPS to determine the electronic environment of the catalysts and the results also confirmed the existence of vanadium species. The XPS spectrum of the sample is shown in Figure 5c, where V, O, C, and Al were detected on the catalysts. The corresponding V 2p, O1s, and Al 2p of the XPS core-level spectra are shown in Figures 5d–5f. As can be seen in Figure 5d, V 2p3/2 and V 2p1/2 peaks were present in the V 2p spectrum due to the spin-orbit splitting of the P orbital. The deconvolution of V 2p3/2 presented 3 main components at 516.0 eV, 517.5 eV, and 518.9 eV, which can be assigned to V3+ , V4+ , and V5+ , respectively [34,35]. The presence of V3+ and V4+ may have been responsible for the high catalytic activity and stability of the catalyst, since V3+ is the main active species and V4+ has better resistance to coke formation in a reaction [5,36]. The peak of O1s at 532 eV was related to the O-V bond, and the peak at 536 eV contributed to the C-O, C=O, and C-OH bonds [37]. Meanwhile, Figure 5f shows the high-resolution XPS spectrum of Al 2p. The peaks at 71.9 eV and 75.8 eV can be ascribed to the Al-O and Al-OH bonds, respectively [38]. Among them, the existence of a high proportion of Al-OH indicated that a large amount of hydroxyl groups were located on the surface of the catalyst, which was advantageous for increasing the catalytic activity of the catalyst [5]. Although the XPS spectrum showed the presence of vanadium, no obvious particles of vanadium oxide could be observed in the TEM image, indicating that vanadium oxide should be present at a size less than 1 nm or should form a solid solution with the support [39]. Thus, the vanadium species may have been anchored to the surface of the support in an oligomer state.

### 3.6. Reaction-regeneration cycles

**Figure 6:**
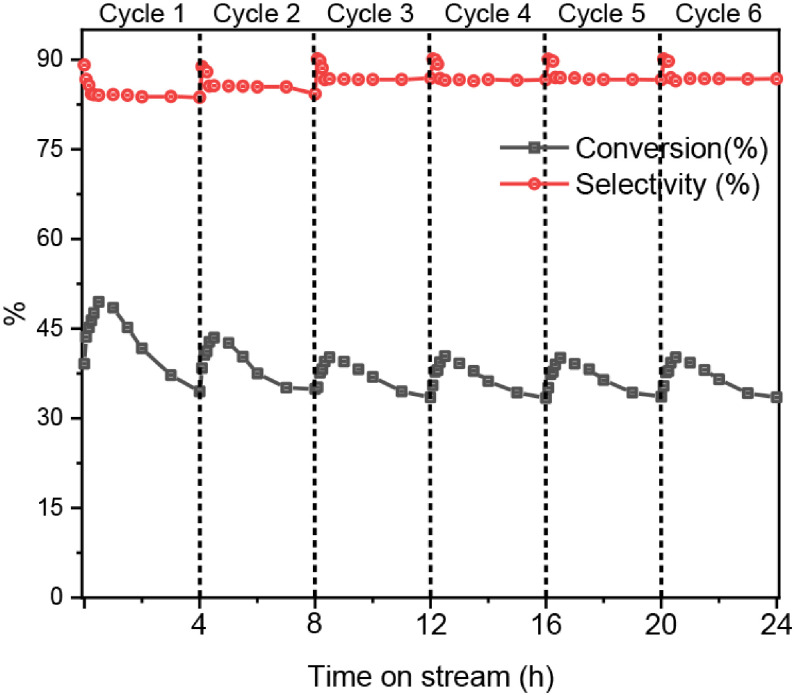
Propane conversion and propylene selectivity of reaction-regeneration cycles over the O-10-VO _x_ /Al _2_ O _3_ catalyst.

In order to investigate the stability and reusability of the as-prepared catalysts, the O-10-VO _x_ /Al _2_ O _3_ catalysts were tested via reaction-regeneration cycle experiments for PDH. The catalysts were regenerated in situ at 580 ◦ C for 2 h in an air flow of 30 mL/min. After 4 h of PDH reaction, the flow was then switched to a N _2_ flow of 30 mL/min for 1 h. As shown in Figure 6, the initial activity of the catalysts decreased over time in the first cycle due to deactivation of the catalysts caused by the formation of carbon. Subsequently, in the second cycle, it can be seen that the activity slightly decreased and the conversion of propane decreased slightly by about 5%, while selectivity of propylene increased by about 4%. The irreversible decrease of catalytic activity occurred in the second cycle, possibly because of the texture changes of the catalysts and the aggregation of the VO _x_ species [4]. After the first regeneration, the activity of the catalysts remained stable (propane conversion was between 43% and 48%, propylene selectivity was between 84% and 90%). This meant that the structure of the VO _x_ species and porous alumina had been stabilized.

### 3.7. Conclusions

The facile 1-pot synthesis of VO _x_ /Al _2_ O _3_ catalysts with different vanadium contents for the significant enhancement of catalytic performance and stability for PDH was presented herein. These catalysts were highly efficient, cost-effective, and environmentally friendly, and vanadium is a low-polymerized VO _x_ species, which is the main active species for catalytic dehydrogenation. In addition, the O-10-VO _x_ /Al _2_ O _3_ catalysts exhibited optimized catalytic performance. The obtained VO _x_ /Al _2_ O _3_ catalysts had the following advantages: 1) Catalysts prepared by the 1-pot method had higher specific surface area and pore volume, which can increase the access between propane and the active sites. 2) The catalysts had higher catalytic performance than the VO _x_ /Al _2_ O _3_ catalysts prepared by the IWI method. 3) The active sites were well dispersed and stable because the vanadium precursor and aluminum precursor were mixed in solution, and the introduction of CMC enhanced the interaction between the vanadium and aluminum during the assembly process.

Supplementary Materials
