# Supplementary material for: One-pot synthesis of VO x /Al 2 O 3 as efficient catalysts for propane dehydrogenation
Source: Turk J Chem. 2020 Feb 11;44(1):112–24. doi: 10.3906/kim-1907-53 (PMC7751822; doi:10.3906/kim-1907-53)
Supplement: Supplementary file 1 — Supplementary Materials [file turkjchem-44-112-sup001.pdf]

## Supplemental information

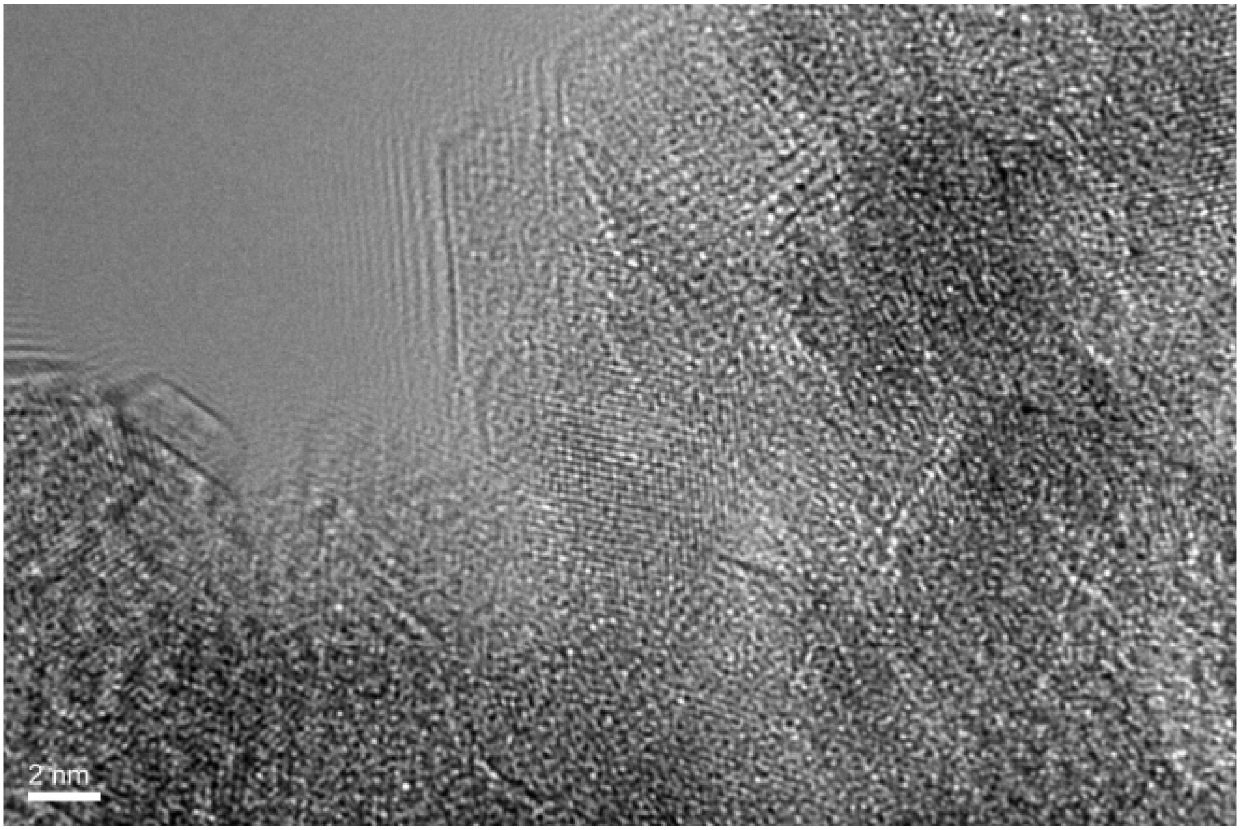


**Figure S1**. High-magnification TEM image of O-10-$VO_x/Al_2O_3$.

**Table S1**. TOF value and propylene selectivity over the vanadium-based catalysts in PDH.

| Catalysts | V (wt.%) | TOF $\times 10^{-3}$ ($s^{-1}$) | Propylene selectivity (%) |
|---|---|---|---|
| O-8-$VO_x/Al_2O_3$ | 8 | 2.7 (1 h[a]); 1.7 (4 h[b]) | ~ 85 |
| O-10-$VO_x/Al_2O_3$ | 10 | 3.1 (1 h); 2.1 (4 h) | ~ 84 |
| O-12-$VO_x/Al_2O_3$ | 12 | 2.1 (1 h); 1.6 (4 h) | ~ 80 |
| I-8-$VO_x/Al_2O_3$ | 8 | 2.9 (1 h); 2.0 (4 h) | ~ 81 |
| I-10-$VO_x/Al_2O_3$ | 10 | 2.8 (1 h); 1.8 (4 h) | ~ 81 |
| I-12-$VO_x/Al_2O_3$ | 12 | 2.2 (1 h); 1.4 (4 h) | ~ 77 |

[a] Initial 1 h of reaction, [b] Initial 4 h of reaction.
